# Biofilm-Innate Immune Interface: Contribution to Chronic Wound Formation

**DOI:** 10.3389/fimmu.2021.648554

**Published:** 2021-04-09

**Authors:** Zoya Versey, Waleska Stephanie da Cruz Nizer, Emily Russell, Sandra Zigic, Katrina G. DeZeeuw, Jonah E. Marek, Joerg Overhage, Edana Cassol

**Affiliations:** ^1^ Department of Health Sciences, Carleton University, Ottawa, ON, Canada; ^2^ Department of Complex Continuing Care, Saint Vincent Hospital, Ottawa, ON, Canada; ^3^ Centre for Infection, Immunity and Inflammation, University of Ottawa, Ottawa, ON, Canada

**Keywords:** chronic wound, delayed healing, innate immune responses, inflammation, biofilm, host-pathogen interaction, skin microbiome

## Abstract

Delayed wound healing can cause significant issues for immobile and ageing individuals as well as those living with co-morbid conditions such as diabetes, cardiovascular disease, and cancer. These delays increase a patient’s risk for infection and, in severe cases, can result in the formation of chronic, non-healing ulcers (e.g., diabetic foot ulcers, surgical site infections, pressure ulcers and venous leg ulcers). Chronic wounds are very difficult and expensive to treat and there is an urgent need to develop more effective therapeutics that restore healing processes. Sustained innate immune activation and inflammation are common features observed across most chronic wound types. However, the factors driving this activation remain incompletely understood. Emerging evidence suggests that the composition and structure of the wound microbiome may play a central role in driving this dysregulated activation but the cellular and molecular mechanisms underlying these processes require further investigation. In this review, we will discuss the current literature on: 1) how bacterial populations and biofilms contribute to chronic wound formation, 2) the role of bacteria and biofilms in driving dysfunctional innate immune responses in chronic wounds, and 3) therapeutics currently available (or underdevelopment) that target bacteria-innate immune interactions to improve healing. We will also discuss potential issues in studying the complexity of immune-biofilm interactions in chronic wounds and explore future areas of investigation for the field.

## Introduction

Wounds are a broad category of injuries that include everything from minor cuts and scrapes to surgical incisions and serious trauma. In healthy individuals, minor wounds heal quickly without complication. However, larger wounds take more time to heal, increasing risk of infection. Delayed wound healing is a significant issue among ageing individuals, those with immobility and those with chronic diseases such as diabetes, vascular disease, and cancer (1–3). In the most severe cases, these delays can result in the formation of non-healing or chronic ulcers, which can cause significant pain, prolonged hospitalization, loss of function, and may eventually lead to amputations and/or the development of sepsis (1, 4). Wound care (acute and chronic) is labor-intensive and represents a substantial economic burden on healthcare systems, costing billions of dollars annually in North America (5). As the global population continues to age and experience increasing rates of co-morbid chronic diseases such as diabetes, there is an urgent need to understand the pathophysiology of delayed healing or the formation of non-healing wounds to develop more effective therapies that can repair tissue damage and restore health (3).

There is an increasing interest to understand how dysregulated host-pathogen interactions affect healing processes. For instance, colonization of the wound bed with low levels of bacteria does not affect healing (6). However, local infection with high levels of replicating bacteria plays a significant role in delayed healing and in non-healing ulcer formation (6, 7). Chronic wounds show a considerable diversity in the bacterial species at the site of injury, but it is unclear how these differences contribute to chronicity. Further, these bacteria form robust biofilms, which embed the bacterial cells in a self-produced polymeric matrix, protecting them from host immune responses and antibiotics. This structure provides numerous advantages to the community such as metabolic cooperation, passive resistance, and horizontal gene transfer (8). It has also been shown to impair the tissue repair processes and promote a low-grade inflammatory response (7, 9). In this review we will discuss the current understanding of how interactions between bacterial biofilms and innate immune cells drive damaging inflammatory processes that contribute to delayed healing in chronic wounds. We will also discuss how we can target these interactions to develop novel therapeutics for individuals with difficult to treat chronic wounds.

## Contribution of Bacterial Biofilms to Chronic Wounds

### Overview of Bacterial Biofilms

Bacteria exist as single, planktonic cells or as multicellular communities and aggregates with or without surface attachment, called biofilms (10–12). Biofilms possess distinct characteristics compared to planktonic cells, including increased antibiotic tolerance, changes in gene expression, and altered host interactions (13). Bacteria within these structures are embedded in a self-produced extracellular polymeric substance (EPS) composed of extracellular DNA, proteins, exopolysaccharides and water. In addition to the microbial components, the EPS can also include host substances such as proteins, DNA, immunoglobulins, and blood components (14, 15).

Biofilms are complex and diverse structures that can be composed of single species or can be polymicrobial (15). They are up to 1,000 times more tolerant to antimicrobial agents and disinfectants than planktonic cells (16). Further, the immune system is often inefficient in combating biofilm-related infections (17, 18). Several factors contribute to the increased robustness of biofilms including low growth rates, high cell density, the presence of persister cells, nutrient and oxygen gradients, horizontal gene transfer, efflux pumps, and high rates of mutation (16, 19). The presence of the EPS matrix is also considered a physical barrier against antimicrobial agents and the host immune responses since it reduces the diffusion of drugs, antibodies and immune cells into the biofilm.

Biofilm-growing bacteria have been shown to colonize medical devices (*e.g.*, contact lenses, cardiovascular valves, implants, ortho-dental prosthetics, urinary and central vascular catheters) and a variety of host tissues, causing many chronic infections, including osteomyelitis, vaginosis, lung infections in cystic fibrosis patients, ventilator-associated pneumonia, device-related infections, and chronic wound infections (15, 20). It is estimated that bacteria in biofilms cause up to 80% of all human infections (21) and are involved in more than 60% of all chronic wound infections (15). These biofilms are composed of bacterial species found in the normal flora of the skin, the gut and oral mucosa as well as in external environments (22).

### Human Skin Microbiome

The human body is naturally colonized by thousands of different microbial species that collectively form a complex ecosystem called the human microbiome (23). More than 1,000 different bacterial species can be found on the human body, and it is estimated that there are up to 150 times more microbial genes than human genes within the human body (24, 25). These microorganisms selectively colonize different parts of the body, such as the skin, gastrointestinal tract, conjunctiva, oral cavity, vagina, uterus, and lungs (26). The human microbiome plays essential roles in health, including protection against invading pathogens, metabolism of molecules, nutrient acquisition, control of cellular proliferation and differentiation, and development of the immune system (25, 27, 28). However, the composition and diversity of the microbiome can be altered by several factors (e.g., diet, use of antibiotics, mode of birth, and age) and it can become associated with opportunistic pathogens and specific isolates that cause severe infections (24, 28). For example, certain isolates of *Staphylococcus aureus* can be either commensal, opportunistic or pathogenic.

The highest number of microbes are found in the colon (10^14^ bacteria), followed by the skin (10^12^ bacteria) (26). The skin microbiome is highly adapted to the skin’s physiological environment, such as the absence of several nutrients, an acidic pH and temperature. The bacterial microbiota found in each skin region depends on the microbe’s ability to thrive in these conditions. For example, bacteria from the *Staphylococci* genus use urea from the sweat as a nitrogen source whereas *Cutibacterium acnes* (formerly *Propionibacterium acnes*) produce fatty acids by metabolizing triglycerides present in sebum (27, 29). The skin is naturally considered a physical barrier against external stressors, and the natural skin microbiota protects the body by competing with pathogenic microbes and impairing their development, catabolizes natural skin products, such as lipids, and modulates the immune system (29). If the skin is disrupted, or there is an imbalance between pathogenic and the natural microbiota and infections can occur (27, 29).

**Figure 1:**
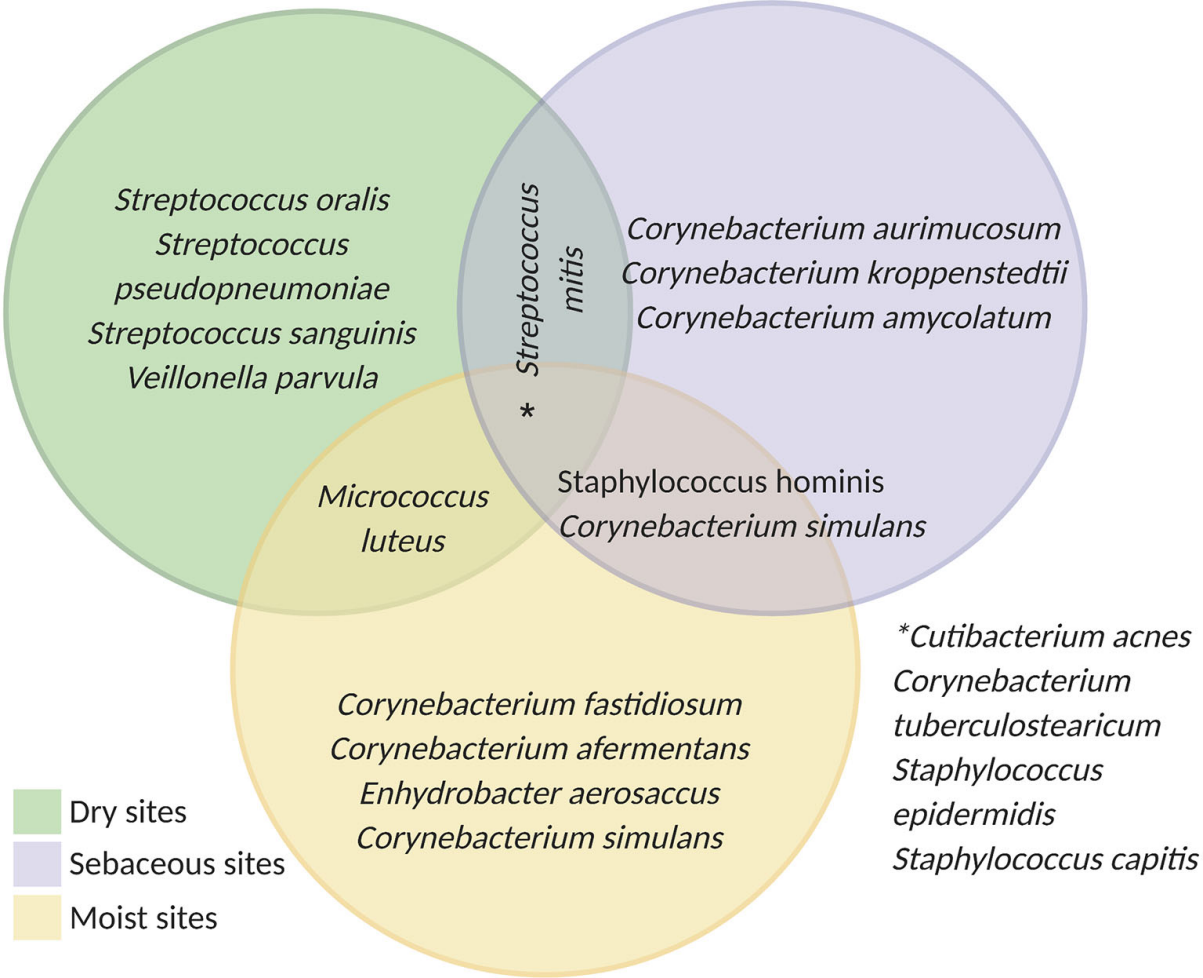
Schematic of skin microbiota according to the physiological sites: dry (green): buttock, volar forearm, hypothenar palm; moist (yellow): plantar heel, popliteal fossa, toe web space, axillary vault, and nare; sebaceous (purple): back, occiput, retroauricular crease, and glabella. Developed using data from (27).

Overall, most microbes from the skin microbiota belong to four different phyla: Actinobacteria, Firmicutes, Bacteroidetes, and Proteobacteria (29). While certain species are found in multiple microenvironments (e.g., *C. acnes*, *Staphylococcus epidermidis*, and *Staphylococcus capitis*), the composition of the skin microbiome differs significantly according to the environmental conditions and location. **Figure 1** highlights some of the most common bacterial species found in dry, sebaceous and moist environments. Dry areas represent the most diverse microbial environments. In these regions, streptococcus *s*pecies are very common but actinobacteria, proteobacteria, firmicutes, and bacteroidetes are also detected including *Corynebacterium tuberculostearicum*, *Staphylococcus* sp., *Veillonella parvula* and *Micrococcus luteus* (27, 30). In moist and sebaceous sites corynebacterium are highly prevalent. However, these microenvironments are also populated with other common species including *Staphylococcus hominis*, *Enhydrobacter aerosaccus*, *Streptococcus mitis* and *Micrococcus lutues* (27, 29, 30).

It is important to acknowledge, that while bacteria are the most prevalent microorganisms found on the skin, fungal species also contribute to the microbiota (27). *Malassezia* is among the most common fungal genera accounting for 53-80% of the fungal population (29). Findley et al. found that *Malassezia* is found on several sites including retroauricular crease, nare, palm, back, and volar forearm. Other fungal species were also detected, with the plantar heel showing the greatest diversity with approximately 80 genera, including *Malassezia, Aspergillus, Cryptococcus, Rhodoturulla, Epicoccum* (31).

### From Contamination to Infection

The human skin microbiota can influence the wound healing process and affect the severity of infections. In chronic wounds, microbial contamination and colonization can evolve to an infection, a process that involves different microorganisms (32). Contamination refers to the presence of non-proliferating bacteria originating from the natural microbiota or the environment. All wounds are contaminated by microorganisms; however, if they encounter favorable conditions that support survival, the wounds will become colonized. Colonization is characterized by the presence of multiplying microorganisms on the surface of the wound (33–35). In these initial processes, the host immune defenses are not triggered, and there are no overt clinical signs of infection. This is likely because the wound is colonized by Gram-positive bacteria, especially those belonging to the *Staphylococci* genus, which do not elicit strong immune responses (32, 36).

In the later stages, Gram-negative bacteria, mainly rod-shaped bacteria such as *Pseudomonas* sp., *Escherichia coli, Klebsiella pneumoniae*, and *Enterobacter* spp. contaminate the wound and become the predominant species in this microenvironment. These species typically originate from the urogenital tract of the patient or from the nearby environment. Colonization of may also be affected by any antibiotic use (local or systemic). This acute colonization is an intermediate stage before deeper tissue infections and only elicits a localized immune reaction (32, 37). In this stage, the bioburden increases, and the presence of these microbes can delay the healing process. Finally, the last step is infection, in which the microorganisms invade the deep tissues, the body cannot control the levels of microbes, and an intense host response is induced (32, 36). This stage is also characterized by the rapid consumption of oxygen by aerobic bacteria, which favors anaerobic microbes (32, 33).

### Bioburden

Many aspects of wound bioburden correlate with healing outcomes. These include microbial load, microbial diversity, and presence of pathogenic organisms (38). Microbial load is commonly used to diagnosis a wound infection when clinical manifestations are absent (38). While the skin microbiota contains approximately 1 billion bacteria/cm^2^ of tissue (39, 40), a microbial load higher than 10^5^ colony-forming units (CFU) per gram of tissue is generally considered the standard reference for diagnosing infection (41). However, this value can vary according to the type of infection and type of wound evaluated. In diabetic foot ulcer (DFU), the microbial load may be higher than in venous leg ulcer (VLU) patients (38, 41).

### Microbial Diversity

Microbial composition and diversity are other factors associated with delayed healing. The most common bacteria found in the wound bed are aerobes such as *Staphylococci ssp.*, *Corynebacterium spp*, *Pseudomonas* spp. as well as anaerobes such as *Anaerococcus spp* and *Finegoldia* spp (42–45). However, a higher diversity of microbial species in the wound bed can be associated with impaired healing outcomes (38). In addition to overall diversity, the presence of specific pathogenic organisms or even certain isolates can also contribute to chronic wound formation. Some strains of *S. aureus* and group A streptococci, have been shown to cause severe infections and trigger an intense inflammatory response, affecting the healing process (32). Moreover, high levels of *S. aureus* and *P. aeruginosa* can affect healing outcomes because these organisms are often resistant to different classes of antibiotics, they form biofilms with antiphagocytic activities and they produce several virulence factors, including secretion of toxins and enzymes, which drive further tissue damage (34, 46). Further studies are required to understand how single species vs. poly-microbial biofilms may contribute to these processes.

### Methodologies for Characterization

Several factors must be taken into consideration when studying the skin and wound microbiota. Among the most important, are the sample collection technique and the method used to characterize the microbial population (*i.e.*, culture-based or molecular methods) (47). At the level of sample collection, wound swabs and tissue biopsies (or debridement tissue) are commonly used for microbiota analysis. Swabbing the wound surface is often preferred because it is non-invasive, and it can be performed multiple times for longitudinal studies. However, dry swabs generally collect a low biomass and do not capture the diversity of species found in deeper tissues (48). Nakatsuji et al. found the bacterial diversity on the surface was not the same as that in the sub-epidermis, which included high levels of proteobacteria (49). Further, Travis et al. found there was minimal correlation between the number and types of bacteria found in swabs compared to tissue biopsies. They also found tissue biopsies contained an overall greater diversity of bacterial species but that the frequency of potential pathogens was higher in wound swabs (50). While biopsies represent the gold standard to capture the true diversity of bacteria found in skin and wound samples, it is invasive and requires a skilled clinician.

The two techniques most widely used to characterize bacteria in chronic wound infections are: A) culture-based methods, in which the microbial culture is collected from the affected tissue and bacteria are grown on selective or nonselective media; and B) molecular-based methods, in which the bacterial 16S rRNA is sequenced and microbes are identified based on databases (41, 51–53). While the culturing of bacteria is primarily used in the hospital setting, this method is usually limited to growing certain strains of bacteria and does not allow for the detection of fastidious and slow-growing bacteria, viable but nonculturable bacteria (VBNC) or dormant bacteria (12). The use of molecular methods, such as RNA sequencing, has increased in recent years but is still predominantly used for research. In comparison to culturing methods, these approaches allow for the identification of a larger diversity bacterial species in the wound. It can also be used to elucidate microbial activities, behaviors, strategies, and processes during infections (12). However, RNA sequencing is associated with a substantial demand for financial, time and bioinformatic support. Moreover, it cannot distinguish between living, dead or dormant bacteria and might overlook minority species (52). To complement these approaches, microscopy can be used to capture the complexity and the organization of bacterial populations in the wound environment. It can be used to visualize and characterize individual cells within a population/structure (e.g., biofilm) and provide important insights into their interactions and structural organization (48, 54). Regardless of the assay used, several other factors impact the diversity (or our ability of evaluate the diversity) of the wound microbiota including patient demographics, personal hygiene, grade of wound severity, patient’s immune status, and ongoing or previous use of antimicrobial therapies (12, 52).

### Bacterial Diversity Across Chronic Wound Types

Chronic wounds or non-healing wounds are commonly defined as wounds that have failed to proceed through the normal phases of wound healing in an orderly and timely manner (55). The most common types of these wounds are diabetic foot ulcers, pressure ulcers, venous leg ulcers, abscesses and surgical site infections. While bacteria contribute to the pathophysiology of delayed healing and chronic wound formation, the molecular and cellular mechanisms underlying these processes remain incompletely understood. To date, most studies have been cross-sectional nature (**Table 1**). Few have used longitudinal approaches to evaluate how bacterial communities change in different chronic wounds over the course of infection. Moreover, fewer studies have investigated correlations between the microbiota and specific wound outcomes (53).

**Table 1:** Summary of most common bacterial species found in chronic wounds (excluding DFU).

Venous Leg Ulcers				
Gram-positive Aerobes	Gram-negative Aerobes	Gram-positive Anaerobes	Gram-negative Anaerobes
*-Actinotignum schaalii* *-Alcaligenes faecalis* *-Brevibacterium casei* *-Corynebacterium amycolatum* *-C. jeikeium* *-C. simulans* *-C. striatum* *-C. tuberculostearicum* *-Enterococcus faecalis* *-Staphylococcus aureus* *-S. epidermidis* *-S. haemolyticus* *-S. lugdunensis* *-S. pettenkoferi* *-Streptococcus agalactiae*	*-Achromobacter xylosoxidans* *-Acinetobacter baumannii* *-Citrobacter* spp*. * *-Delftia acidovorans* *-Enterobacter cloacae* *-E. hormaechei* *-Klebsiella oxytoca* *-Proteus* spp. *-Pseudomonas aeruginosa* *-P. fluorescens* *-Serratia nematodiphila* *-Stenotrophomonas maltophilia*	*-Anaerococcus vaginalis* *-Finegoldia magna* *-Peptoniphilus harei* *-Peptostreptococcus assacharolyticus* *-Cutibacterium acnes*	*-Bacteroides tectus* *-Flavobacterium succinicans* *-Fusobacterium gonidiaformans*
**Pressure Ulcers**
**Gram-positive aerobes**	**Gram-negative aerobes**	**Gram-positive Anaerobes**	**Gram-negative Anaerobes**
*-C. jeikeium* *-C. striatum* *-C. tuberculostearicum* *-E. faecalis* *-S. aureus* *-S. epidermidis* *-S. haemolyticus* *-S. lugdunensis* *-S. agalactiae* *-S. dysgalactiae*	*-A. baumannii* *-D. acidovorans* *-E. hormaechei* *-Escherichia* spp. *-P. mirabilis * *-P. aeruginosa* *-Serratia* spp. *-S. maltophilia* *-*unclassified *Enterobacteriaceae* spp.	*-Allobaculum* spp. *-A. vaginalis* *-Eubacterium dolichum* *-F. magna .* *-Peptococcus* spp. *-Peptoniphilus ivorii*	*-B. fragilis* *-Dialister* spp. *-F. nucleatum* *-Prevotella bivia*
**Surgical Site Infections**
**Gram-positive aerobes**	**Gram-negative aerobes**	**Gram-positive Anaerobes**	**Gram-negative Anaerobes**	**Other**
*-Bacillus* spp. *-E. faecalis* *-*Coagulase-negative *staphylococci* (CoNS) *-C. striatum* *-C. tuberculostearicum* *-Granulicatella elegans* *-*methicillin-resistant *S. aureus* (MRSA) *-S. aureus* *-S. epidermidis* *-S. haemolyticus* *-S. lugdunensis* *-S. agalactiae* *-S. mitis* *-S. salivarius*	*-A. baumannii* *-A. lwoffii* *-D. acidovorans* *-Diaphorobacter* spp. *-K aerogenes* *-E. cloacae * *-Enterobacteriaceae* spp. *-E. coli* *-K. oxytoca* *-K. pneumoniae* *-Moraxella* spp. *-Morganella morganii* *-Neorhizobium* spp. *-Novosphingobium spp.* *-Paracoccus* spp. *-P. mirabilis* *-P. aeruginosa* *-Ralstonia pickettii* *-S. nematodiphila* *-Sphingomonas* spp. *-S. maltophilia*	*-A. vaginalis* *-Clostridium* spp. *-F. magna* *-C. acnes*	*-Cloacibacterium* spp. *-F. nucleatum* *-Methylobacterium* spp.	*-Candida albicans*
**Abscesses**
**Gram-positive aerobes**	**Gram-negative aerobes**	**Gram-positive Anaerobes**	**Gram-negative Anaerobes**	
*-*CoNS *-C.accolens/gurimucosum* *-C. afermentans* *-C. mucifaciens* *-C. tuberculostearicum* *-Enterococcus* spp. *-Micrococcus luteus * *-*MRSA *-*Methicillin sensitive *S. aureus* (MSSA) *-S. aureus* *-S. caprae/capitis* *-S. epidermidis* *-S. haemolyticus* *-S. lugdunensis* *-S. petrasii* *-S. agalactiae*	*-Chryseobacterium* spp. *-Haematobacter massiliensis* *-P. aminovorans* *-P. versutus* *-Proteus* spp. *-Rhodanobacter* spp. *-Sphingomonas* spp.	*-F. magna* *-C. acnes*	*-Flavobacterium* spp. *-Porphyromonas* spp. *-Prevetolla* spp.

### Diabetic Foot Ulcers

To date, most studies analyzing microbial communities in chronic wounds have focused on infections from patients with diabetic foot ulcers. These investigations have been recently reviewed in significant detail and are beyond the scope of the current review (52, 56–59). Briefly, the most frequently identified genera in DFU include Staphylococcus, Corynebacterium, Pseudomonas, Streptococcus, Stenotrophomonas, Enterobacter, Escherichia, Enterococcus, Serratia, Acinetobacter, Peptoniphilus, Anaerococcus and Finegoldia (42–45, 50, 60–67). The most common pathogens found were the Gram-positive bacteria *S. aureus*, *S. epidermidis*, *Enterococcus faecalis* and *Streptococcus agalactiae* and the Gram-negative bacteria *P. aeruginosa*, *Stenotrophomonas maltophilia*, *E. coli* and *Acinetobacter baumannii* (43–45, 62).

### Other Types of Chronic Wounds

The bacterial communities of other types of wounds, including venous leg ulcers, pressure ulcers, surgical site infections, and abscesses, have not been fully evaluated. Previous studies have shown that like DFU, commonly identified genera include Staphylococcus, Corynebacterium, Streptococcus, Enterococcus, Pseudomonas, Stenotrophomonas, Enterobacteriaceae, Acinetobacter and Finegoldia (**Table 2**, and (43, 60, 78–82). To date, Wolcott et al. have performed one of the largest studies collecting samples from 916 venous leg ulcers, 767 decubitus ulcers, and 370 samples from nonhealing surgical wounds and evaluating bacterial diversity using 16s rDNA sequencing. They found that the most frequent bacterial species found across all wound types were *S. aureus*, *S. epidermidis*, *E. faecalis*, *P. aeruginosa*, *S. maltophilia* and *Finegoldia magna*. They also observed a high prevalence of anaerobic bacteria in wound samples, including *Finegoldia* spp. and *Anaerococcus* spp (present in 24% and 25% of wounds, respectively) and variety of commensal bacteria including Staphylococci and Corynebacteria across wound types (43). Interestingly, unlike DFU, these wounds contained *Cutibacterium acnes* and other Cutibacterium species suggesting an important role for the location of the wounds in spectrum of microbial colonization (43, 83, 84). Further, most of these infections were polymicrobial biofilms consisting of many different species. Only 7% of wound samples were found to be mono-species biofilms with *P. aeruginosa* and *S. epidermidis* being the most common bacteria growing in single-species biofilms in wounds (43). More *in vivo* studies with larger samples sizes are urgently needed to fully understand the importance of microbial diversity, biofilms and the wound microbiome in chronic wounds infections and to elucidate the impact of aerobic, anaerobic, pathogenic and commensal bacteria in driving chronic immune activation and inflammation in situations of delayed wound healing.

**Table 2:** Summary of studies characterizing host immune responses to bacteria and bacterial biofilms in wound models.

Wound Model	Bacterial Species/Component	Host Response	Effects on Wound Healing	Reference
Mouse full-thickness excisional wound	*P. aeruginosa* PAO1	Early infection. In skin: ↑ neutrophils, ↓ NK cells, ↓CD11b^+^ DCs, ↓Gr1-low MoDCs. In spleen: ↓T-cells. In lymph nodes: ↑pDCs. Late infection. In spleen: ↑macrophages, ↓NK cells, ↓IKDCs. In lymph nodes: ↑pre-apoptotic T-cells, ↑pDCs. Planktonic or biofilm infection: ↑TNF, ↑CXCL1, ↑IL-6, ↑IL-1b mRNA expression in skin HK infection: acute ↑neutrophils, ↓CD103^+^ DCs	↑Bioburden of biofilm-infected wounds compared to planktonic infection	(68)
Kostelec minipig excisional flank wound reaching subcutaneous fat	*S. aureus, E. faecalis, B. subtilis, P. aeruginosa* clinical isolates Preformed biofilm	↑IL-8, ↑CXC-13, ↑arginase-1 ↑oxidative stress response (superoxide dismutase 2, angiopoietin-like 4) ↑MMP-1, ↑MMP-3 ↓collagen-1, ↓laminin-2	↓Granulation tissue formation	(69)
Pathogen-free mouse burn-induced wound	*P. aeruginosa* PAO1 embedded in seaweed alginate to mimic biofilm	↑ IL-1β, ↓AMP S100A8/A9 ↓KC, ↓G-CSF ↓VEGF	↓Wound closure	(70, 71)
Mouse full-thickness excisional wound	Absence of commensal microbiota	↑TNF-α, ↑ IL-10 ↑Alternatively activated macrophage (Dectin-1, Mannose receptor-1, Fizz-1, and Arginase-1) infiltration. ↑Mast cell infiltration ↓Neutrophil infiltration ↑ VEGF, ↑ type III collagen, ↑TGF- β1	↑Wound closure ↑Angiogenesis ↓Scar tissue	(72)
New Zealand white rabbit full-thickness ear wound	*P. aeruginosa* PAO1 *S. aureus* UAMS-1 Polymicrobial biofilm	↑IL-1β, ↑TNF-α mRNA expression compared to single-species biofilm	↓Epithelial and granulation tissue formation	(73)
New Zealand white rabbit full-thickness ear wound	*S. aureus* UAMS-1 Planktonic and biofilm	Low-grade, chronic inflammation (↓IL-1β, ↓TNF-α) mRNA expression compared to planktonic infection	↓Wound healing	(74)
Diabetic mouse full-thickness excisional wound	Wound microbiota	Longitudinal transcriptional shift in wound microbiota correlates with impaired and prolonged host defense response	↓Wound healing	(75)
Mouse full-thickness wound	Bioluminescent *S. aureus* SH1000	↑Systemic and wound infiltrating PMNs	No significant delay in wound healing	(76)
Surgical biopsy of patients with local infection due to a splinter, a bite, an abscess, or thrombophlebitis N=5	*S. aureus* was present in all skin biopsies	↑Granulocytes, ↑T-cells, ↑monocytes/macrophages in skin ↓IL-8, ↑IL-6 ↑E-selectin, ↑VCAM-1 Keratinocytes: ↑ICAM-1, ↑TNF-α, ↑IL-1α	NA	(77)

NA, not applicable; NK, natural killer cells; DC, dendritic cell; MoDC, monocyte-derived DCs; pDC, plasmacytoid DC; IKDC, interferon killer DCs; HK: heat-killed; PMN, polymorphonuclear leukocytes; MMP, matrix metalloproteinase; AMP, antimicrobial peptide; KC, keratinocyte-derived chemokine; G-CSF, granulocyte-colony stimulating factor; VEGF, vascular epithelial growth factor.

## Contribution of Chronic Innate Immune Activation and Inflammation to Chronic Wounds

### Overview of Wound Healing

**Figure 2:**
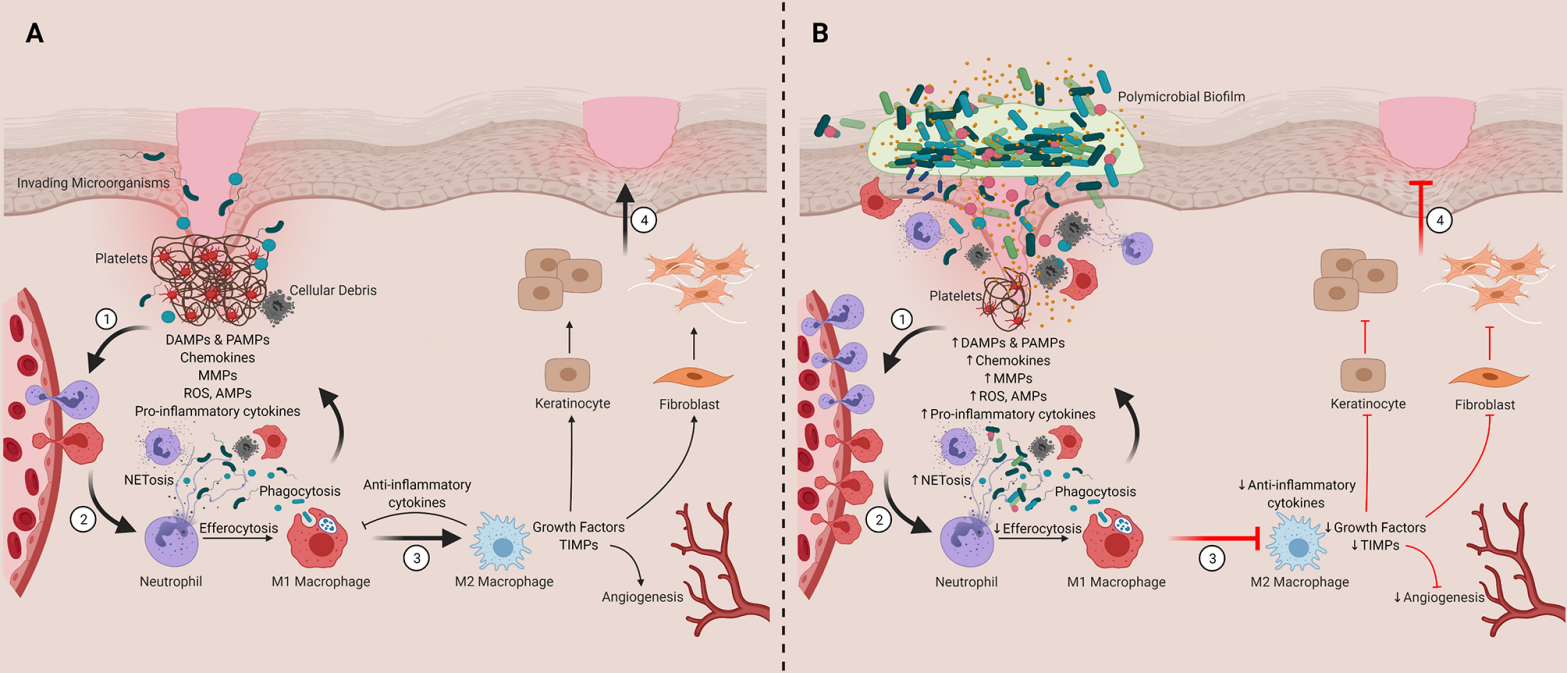
Contribution of innate immune cells and inflammation to timely and delayed wound healing. **(A)** Representation of the four phases of wound healing ([1] Hemostasis, [2] Inflammation, [3] Proliferation and [4] Tissue Remodeling). **(B)** Chronic wounds are stalled in the inflammatory stage. We hypothesize that this inflammation is sustained by chronic activation of the innate immune system, which is driven their interactions and responses to polymicrobial biofilms found in and on the wound bed. DAMPs, damage-associate molecular patterns; PAMPs, pathogen-associated molecular patterns; MMPs, matrix metalloproteinases; ROS, reactive oxygen species; AMPs, antimicrobial peptides; TIMPs, tissue inhibitor of metalloproteinases. Created with BioRender.com.

Wound healing has been extensively described elsewhere, and for that reason, we will only briefly summarize the process here (**Figure 2**) (34, 85–92). Normal healing (**Figure 2A**, described as Steps 1-4) is comprised of four coordinated phases: hemostasis, inflammation, proliferation, and tissue remodeling (34). Hemostasis includes platelet aggregation and activation, which initiates the coagulation cascade and the formation of a transient fibrin scaffold (Step 1) (86, 91, 92). During this process, platelet degranulation releases damage-associated molecular patterns (DAMPs), cytokines, chemokines, and growth factors, which accumulate within the scaffold to generate a chemotactic gradient for immune cell infiltration (Step 2) (86, 88, 90). This infiltration is required to clear dead or damaged cells, cellular debris, and any pathogens that colonize the wound bed. It also prepares the wound for the healing phases. The innate immune system plays a critical role in modulating these processes as well as the transition from inflammation to proliferation phases, which includes a transition of inflammatory macrophages (M1) into anti-inflammatory wound healing cells (M2; Step 3) (86, 87). This anti-inflammatory transition activates keratinocytes and fibroblasts in the wound bed where they proliferate and contribute to healing processes (91). Keratinocytes are essential for wound re-epithelialization (86). Fibroblasts deposit collagen to form the extracellular matrix (ECM) or granulation tissue, which replaces the temporary fibrin scaffold (90, 92). During the proliferation stage, angiogenesis also restores tissue vascularity (86). Finally, in the remodeling phase, fibroblasts replace granulation tissue with scar tissue and contraction occurs, resulting in wound closure (Step 4) (34, 86, 88).

### Role of the Innate Immune System in the Inflammatory Phase of Healing

Although the inflammatory phase involves a wide range of immune cells, such as mast cells, dermal dendritic cells (Langerhans cells), and T lymphocytes, innate immune cells such as neutrophils and macrophages play a central role in regulating healing process (85, 91–93). For the purposes of this review, we will focus on these two cell types.

### Neutrophils

Neutrophils play a central role in healing damaged tissues and resolving infections. Early after the initial tissue damage, DAMPs, pathogen-associated molecular patterns (PAMPs), leukotriene B4 (LTB4), and CXCL8 family chemokines induce and augment neutrophil chemotaxis *via* CXCR2 into the wound bed (94–97). As the first cells recruited to the site of injury, neutrophils perform several diverse functions. First, they amplify inflammatory processes by releasing cytokines such as IL-1β, TNF-α, and IL-6 (85, 98) and function to prevent microbial invasion by phagocytosing microorganisms and releasing reactive oxygen species (ROS), proteases, and antimicrobial peptides (99, 100). Further, they undergo NETosis, where they form neutrophil extracellular traps (NETs) made of decondensed chromatin, histones, granular enzymes, antimicrobial peptides and proteases to immobilize and destroy exogenous pathogens (91, 100). They also release serine proteases, such as cathepsin G and elastase, and matrix metalloproteases (MMP), such as MMP2, 8, and 9 that degrade the ECM, facilitating tissue debridement that makes room for additional immune cell influx (92, 99). Neutrophils also contribute to tissue remodeling and repair. Theilgaard-Mönch et al. demonstrated that infiltrating neutrophils activate and induce the transcritption of genes involved in angiogenesis, keratinocyte adhesion, and migration and proliferation of keratinocytes and fibroblasts at the site of injury (98).

### Macrophages

After damage has occurred, resident macrophages become activated by DAMPs and PAMPs in the local microenvironment, initiating the inflammatory response required to initiate the healing process (101, 102). Pro-inflammatory chemokines and cytokines such as CCL2, IFN-γ, and TNF-α recruit neutrophils and then circulating monocytes into the wound (102, 103). In mouse models, the expression of chemokine receptors CCR2 and CX3R1 has been shown to be critical for mediating monocyte recruitment to damaged tissues (104, 105). These infiltrating monocytes differentiate into macrophages, and along with resident macrophages, potentiate the inflammatory and healing processes.

Macrophages display incredible phenotypic plasticity, existing on a spectrum of inflammatory and anti-inflammatory activation states (106, 107). During the inflammatory phase of wound healing, macrophages display a proinflammatory or a classically activated M1-like phenotype, producing inflammatory mediators such as TNF-α, IL-6, IL-1β, IL-12, IL-23 to facilitate additional leukocyte recruitment and activation (34, 88, 89, 92). M1 cells also work cooperatively with neutrophils to phagocytose damaged cells and clear the wound of bacteria and tissue debris by producing ROS-containing phagosomes (92). Like neutrophils, they also produce MMPs to degrade the wound extracellular matrix to allow for further immune cell infiltration (92, 102). This digested matrix feeds back as DAMPs to amplify inflammatory signaling (108).

Following the clearance of debris and invading microorganisms, macrophages adopt an anti-inflammatory or an alternatively activated M2-like phenotype to support tissue remodeling and repair (106, 107). In vitro studies have shown that IL-4 and IL-13 drives M2 activation and wound healing functions (107). However, *in vivo*, the mechanisms driving this M1 to M2 phenotypic switch may be more complicated and may be regulated by IL-10, glucocorticoids, prostaglandins, miRNAs, and adenosine and peroxisome proliferator-activated receptor (PPAR-γ) signaling in the wound microenvironment (86–88, 109). At the end of the inflammatory phase, macrophage phagocytosis of apoptotic neutrophils (i.e. efferocytosis) may also aid in the transition to the repair phases of wound healing (110).

To initiate tissue remodeling and repair, M2 cells produce IL-10, IL-12, and TGF-β to suppress inflammatory effects of TNF-α and IL-1β (90). During the proliferation phase they also release growth factors, such as vascular endothelial growth factor (VEGF), platelet-derived growth factor (PDGF), and fibroblast growth factor-2 (FGF-2), which promote angiogenesis and keratinocyte and fibroblast proliferation (86, 88, 89, 91, 111). Finally, during the repair and remodeling phase, macrophages stimulate fibroblasts to mature into myofibroblasts that deposit collagen into the wound bed to replace the temporary fibrin scaffold with granulation tissue (86, 92, 112). They also regulate MMPs and tissue inhibitors of metalloproteinases (TIMPs) production to allow for both ECM formation and degradation of excess cellular components to restore homeostasis (92, 113). This M1 to M2 phenotypic switch makes macrophages a central cellular player in regulating the transition from inflammation to proliferation during wound healing (86).

### Dysregulated Inflammatory Processes in Chronic Wounds

In chronic wounds, the orderly process of healing is thought to be stalled in the inflammatory phase and is characterized by persistent activation of the innate immune response (**Figure 2B**, described as Steps 1-4) (55, 87). This sustained inflammation drives additional immune cell infiltration and activation (Step 1), which amplifies MMP, collagenase, and elastase activity and suppresses TIMPs (114–119). This imbalance in proteolytic activity leads to excessive degradation of growth factors, anti-inflammatory cytokines, and ECM components, hindering progression through the phases of healing (120–122). This inflammation also drives excessive NETosis and reactive oxygen production (ROS), which contributes to further tissue damage and impaired healing (Step 2) (123–127). Impaired M1-M2 phenotypic switching has also been implicated in chronic wound formation (Step 3). In venous ulcers, iron overload has been linked to sustained proinflammatory M1 macrophage activation (128, 129). Further, studies in diabetic mice have demonstrated that dysfunctional efferocytosis of apoptotic neutrophils by pro-inflammatory macrophages results in apoptotic cell burden that causes sustained inflammation, preventing macrophages from transition into their anti-inflammatory state (130). In further support of these observations, depletion of M2 macrophages in mice with surgical wounds has been shown to increase neutrophil and M1 macrophage infiltration, which prolonged the inflammatory phase and decreased collagen deposition in wounds (131). Collectively these findings suggest sustained innate immune activation plays a central role driving chronic wound formation. Critically, it is unclear what exactly is driving this chronic innate immune activation. Further, it is unclear if the mechanisms driving this innate immune dysfunction including dysregulated M1-M2 switching differs across tissue microenvironments (e.g., wound types), particularly as emerging data suggests differences between antimicrobial and inflammatory responses across wound types (132)

### Role of Bacteria in Driving Innate Immune Activation and Inflammation in Chronic Wounds

Emerging evidence suggests the wound microbiome and the formation of bacterial biofilms may contribute to delayed wound healing (45, 133–138). However, the causal relationship between microbiome composition, biofilm formation, dysregulated innate immune activation and persistent inflammation in chronic wounds remains poorly understood (12, 139). It is unclear what comes first – if bacteria and biofilm formation drive innate immune dysfunction, or if innate immune dysfunction makes the wound microenvironment more susceptible to biofilm formation. Understanding the role of bacteria-innate immune interactions in driving persistent inflammation and impaired healing in chronic wounds may offer new opportunities to restore healing processes (140). Previous reviews have explored key findings from *in vitro* studies (141–143). Here, we will focus on *in vivo* evidence from animal models and patient samples. We will summarize the existing knowledge on the role of bacteria-innate immune interactions in driving persistent inflammation in chronic wounds and explore novel treatments currently under development to target these interactions.

### General Host Response to Bacteria or Bacterial Components in Wounds

While *in vitro* studies are important for identifying potential cellular and molecular mechanisms underlying innate dysfunction in chronic wounds, these models cannot capture the dynamic and complex nature of the immune response *in vivo*. Animal models and patient samples are better positioned to capture this complexity and can be used to evaluate localized as well as systemic immune responses.

Several studies have shown that wound bacterial infections can alter local and systemic immune responses (**Table 2**). Sweere et al. found that mice with chronic *P. aeruginosa* (PAO1) wound infections showed time-dependent changes in wound, lymph node, and spleen immune cell populations. In this model, early stages were associated with increased skin neutrophil infiltration and a reduction in the number of skin natural killer (NK) cells, CD11b+ dendritic cells (DCs), and Gr1-low monocyte-derived DCs (MoDCs) (68). Similarly, Kim et al. found wound colonization by *S. aureus* increased systemic polymorphonuclear leukocytes (PMN) by twofold and dramatically increased PMN recruitment into the wound bed (76). Systemic immune responses were more pronounced in later stages of wound infection and included increased lymph node plasmacytoid DCs (pDCs), increased splenic macrophages and lymph node pre-apoptotic T-cells and a reduction in NK cells and interferon killer DCs (IKDCs). Based on these findings, authors concluded that adaptive immune responses might not play a significant role in driving the inflammatory response against *P. aeruginosa* (68). In human skin biopsies, Van der Laan et al. found diverse injuries result in different local inflammatory responses (77). Compared to aseptic conditions, wounds infected with *S. aureus* contained increased granulocytes, T-cells, and monocytes/macrophages in the dermal layer and dermis (77). However, further studies are required to understand changes to systemic responses in humans with chronic wounds.

Animals models have also been used to evaluate differences in immune responses to planktonic bacteria vs. biofilm infections in wounds. Sweere et al. found that *P. aeruginosa* infection was associated with increased TNF, CXCL1, IL6, IL1B mRNA expression in mouse skin but that these responses did not differ between planktonic bacteria vs. biofilms, despite the higher bioburden associated with the biofilm structure (68). However, Trostrup et al. found that *P. aeruginosa* biofilms inhibit wound healing by suppressing VEGF, antimicrobial peptide production (S100A8/A9) and neutrophil effector cytokine production (70, 71). In dermal punch wounds in rabbit ears, Gurjala et al. found that S. aureus biofilms trigger lower levels of inflammation in the wound bed compared to planktonic cells. However, they found that the persistent nature of the immune response to biofilms was shown to impair epithelial migration and granulation over time (74). Interestingly, using a similar rabbit model, Seth et al. found that polymicrobial biofilms containing *P. aeruginosa* (PAO1) and *S. aureus* (UAMS-1) triggered substantially higher inflammatory responses compared to single species structures (73). This elevated inflammatory response impaired wound epithelialization and granulation tissue formation. These authors also found that biofilm-deficient mutant strains of *S. aureus* were associated with reduced cytokine mRNA expression suggesting that the biofilm structure may, at least in part, contribute to the inflammatory response (73). In a porcine model, polymicrobial biofilms containing *S. aureus*, *E. faecalis*, *Bacillus subtilis*, *P. aeruginosa* were shown to prolong inflammation, increase necrosis, delay granulation, and impair the development of the extracellular matrix. Gene expression analysis revealed an upregulation of inflammatory mediators such as IL8, CXCL13, and arginase-1 (ARG1), as well as genes associated with oxidative stress response including superoxide dismutase 2 (SOD2) and angiopoietin-like 4 (ANGPTL4) (69).

Several studies have highlighted that the skin commensal microbiota play important functions in the regulation of wound healing and in the innate immune defense against infection (144–146). In fact, using longitudinal transcriptional profiling, Grice et al. found a shift in the wound microbiota of diabetic mice, and found this shift correlated with impaired healing and a prolonged inflammatory response (75). Alternatively, Canesso et al. showed that in germ-free Swiss mice, the absence of commensal microbiota enhanced TNF-α and IL-10 production, infiltration of alternatively activated macrophages and mast cells, and impeded neutrophil infiltration (72). These effects likely contributed to high levels of VEGF, type III collagen, and TGF-β, thereby accelerating wound closure and angiogenesis, and reducing in scar tissue formation (72). Further investigations are required to understand the intricate balance between the pro-wound healing commensal microbiota and pro-wound impairing pathogenic polymicrobial biofilms, as well as their role in driving a dysregulated inflammatory response in chronic wounds in humans.

### Regulators of Immune Responses in Wounds With Bacterial Infections

Several molecules and pathways have been implicated in driving chronic inflammatory responses in non-healing wounds. Most of these have been identified in the context of overt bacterial infections (e.g., abscess) and do not address issues associated with wound chronicity. It is unclear if similar molecules/pathways contribute to delayed healing in wounds with varying levels of bacterial burden (e.g., contamination, colonization, local infection etc.). Despite these limitations, we have summarized these studies below (**Table 3**).

**Table 3:** Summary of molecules and pathways that regulate the inflammatory responses to bacteria in wounds.

Molecule/Pathway	Wound Model	Bacterial Species	Host Response	Effects on Wound Healing	Reference
Leukotriene B_4_ (LTB_4_)/BLT1 activity	Mouse subcutaneous wound	*S. aureus* USA300 LAC	Produced by skin macrophages. ↑Neutrophil chemotaxis (CXCL2, CXCL1, CCL8, CCL4, CCL2, and CXCL1) ↑IFN-γ, ↑IL-12p70 ↑NADPH oxidase bactericidal activity ↓Chronic inflammation (RAGE, TIM, CXCL2, IFN-γ, MMP12, and CCL8)	Organized abscess formation ↑Bacterial clearance	(147)
	Diabetic mouse skin wound (undefined)	*S. aureus* USA300 LAC	↑LTB_4_/BLT1 activity ↑Macrophage and neutrophil infiltration ↓Localization to abscess Early infection: ↑ICAM-1, ↑MCP3, ↑IL-33, ↑IL-12p70, ↑IL-1α, ↑RAGE, ↓CXCL1, ↓CXCL2, ↓MIP1β, ↓CCL2, ↓IL-1β, ↓P-selectin Late infection: ↑CXCL1, ↑CCL2, ↑CCL8, ↑MCP3, ↑MIP1β, ↑P-selectin, ↑ICAM-1, ↑IL-1α, ↑IL-33, ↓IL-12p70, ↓RAGE	↑Abscess size with diffuse immune cell organization ↑Bacterial burden	(148)
Receptor for Advanced Glycation End Products (RAGE)	Mouse subcutaneous wound	*S. aureus* SH1000	↓MPO, ↓MCP-1, ↓HMGB1, ↓IL-6, and ↓TNF-α in skin prior to infection ↓Blood neutrophil and peritoneal macrophage infiltration	Severe open skin lesions ↓Abscess formation ↑Bacterial burden	(149)
Myeloid peroxisome proliferation activator receptor γ (PPARγ)	Mouse subcutaneous wound	*S. aureus* SF8300	For inflammation➔ resolution phase Formation of a glucose-depleted, hypoxic fibrotic abscess	↑Bacterial clearance of established infection that failed to clear during the inflammatory phase	(150)
miR-142	Mouse excisional wound	*S. aureus* NBRC 100910	↑*miR-142-3p* and *miR-142-5p* expression by infiltrating neutrophils and macrophages ↑Neutrophil recruitment and timely phagocytosis	Timely resolution of abscess Protection against horizontal transmission of infection	(151)
Myeloid differentiation primary response 88 (MyD88)	Mouse ear pinna intradermal wound	*S.aureus* Newman	Resident dermal macrophages sense *S.aureus via* myD88 For early recruitment and regulation of PMNs	Timely control and clearance of infection	(152)
IL-33	Patients with abscesses due to MRSA. N=3 Mouse intradermal wound	*S. aureus* CMCC(B)26003	↑IL-33 in human skin samples ↑iNOS in murine skin	↓Lesion size ↓Bacterial burden	(153)
Neutrophil-derived IL-1β/IL-1R signaling	Mouse intradermal wound	*S. aureus* SH1000 ALC2906	Induces expression of genes associated with neutrophil chemotaxis IL-1β is produced by neutrophils. TLR2, NOD2, and FPR1 aid in IL-1β production	↑Abscess formation	(154)
Proline-rich kinase (Pyk2)	Mouse skin abscess. Air-filled pouches in the dermis infected with bacteria	*S. aureus* (unknown strain)	↑PMN activation ↑MPO, ↑MMP9	↑Bacterial clearance	(155)
iNOS	Mouse full-thickness skin incisional and excisional wound	HK polymicrobial culture of *S. aureus*, coagulase-negative *Staphylococcus*, *Enterococcal species, P. mirabilis* previously isolated from normal mouse skin flora	↑IFN-γ from lymphocytes ↑iNOS	NA	(156)

NA, not applicable; MRSA, methicillin resistant S. aureus; MPO, myeloperoxidase; MCP-1, monocyte chemoattractant protein 1 (MCP-1); HMGB1, high mobility group box protein 1; FPR, formyl peptide receptor; iNOS, inducible nitric oxide synthase; PMN, polymorphonuclear leukocytes; MMP, matrix metalloproteinase; HK, heat-killed.

*S. aureus* is the leading cause of skin and soft tissue infections and is often used in abscess infection models (164, 165). Brandt et al. infected mouse skin with methicillin-resistant *S. aureus* (MRSA; USA300 LAC) and found high levels of LTB4 was produced by macrophages surrounding the abscess (147). This inflammatory lipid mediator is required to create a chemotactic gradient that directs neutrophil infiltration and helps to form an organized abscess architecture. It also aids in bacterial clearance by upregulation of pro-inflammatory cytokines (IFN-γ and IL-12p70) and increased NADPH oxidase activity (147) and has been shown to modulate keratinocyte activity (166). However, in diabetic mice, very high levels of LTB4 were found to be associated with dysregulated cytokine production and excessive neutrophil recruitment (148). This dysregulation was found to correlate with large nonhealing lesion areas and increased bacterial loads (148). Further, inhibition of LTB4 signaling through its receptor (BLT1) was shown to restore a functional inflammatory response, suggesting this molecule may play an important role in derailing the inflammatory milieu (148).

Advanced glycation end products (AGEs) and its receptor (RAGE) have also been implicated in the regulation of skin inflammation and diabetic pathologies (167, 168). Interestingly, Na et al. found that RAGE knockout mice infected with *S. aureus* (SH100) experienced less severe skin lesions and increased abscess formation (149). This milder skin damage was associated with increased neutrophil migration and increased bacterial clearance with reduced inflammation (e.g., monocyte chemoattractant protein-1 (MCP-1), high mobility group box protein 1 (HMGB1), IL-6, and TNF-α) (149). Paradoxically, RAGE-/- were also found to have high baseline levels of inflammation prior to infection (149). However, it was speculated that this priming may be protective and help establish rapid innate immune responses in early infection. Collectively, this data suggests that RAGE may be pathogenic in staphylococcal skin infection, particularly in supporting chronic inflammation.

Accumulating evidence suggests appropriate regulation of neutrophil activation is also critical for effective bacterial killing while limiting inflammation. This process is complex and multifactorial. For example, Cho et al. found that neutrophil recruitment and abscess formation is temporally linked to IL-1β/IL-1R activation, which neutrophils produce as part of an autocrine loop. This loop is driven by α-toxin mediated activation of TLR2, NOD2, FPR1 and the ASC/NLRP3 inflammasome (154). Alternatively, Kamen et al. found proline rich kinase 2 (Pyk2) plays an important role in regulating integrin-mediated degranulation responses (155). Further, a number wound healing-related miRNAs have been identified and recent studies suggest that their dysregulation may contribute to wound pathologies (169). Among these, miRNA-142 has been shown to be an inflammation related miRNA that regulates neutrophil recruitment and S. aureus clearance through the inhibition of small GTPase translation (151). Additional studies are required to better elucidate its role in chronic wounds. Interestingly, resident dermal macrophages have been shown to play a central role in regulating both the timely escalation and eventual termination of neutrophil recruitment. Feuerstein et al. showed that this regulation is dependent on MyD88-dependent sensing of *staphylococci* and the recruitment of Ly6Chigh inflammatory monocytes into the skin (152).

In addition to neutrophils, macrophage responses must be tightly regulated during healing processes. In the inflammatory phase, these cells are proinflammatory (M1) and produce high levels of nitric oxide, reactive oxygen species and other antimicrobial peptides, which can be damaging to the local microenvironment. IL-33 represents a potential target as it plays a central role in activating antibacterial responses by activating the AKT-β-catenin pathway, which induces inducible nitric oxygen synthase (iNOS) and increases NO production (153). Alternatively, Mahoney et al. found lymphocyte derived IFN-γ drives the induction of iNOS in mouse wounds infected with heat-killed polymicrobial culture of *S. aureus*, coagulase-negative *Staphylococcus* and *Enterococcal* species as well as *Proteus mirabilis* (156). Alternatively, Xu et al. have shown that decreased NADPH oxidase activity and ROS production is associated with decreased infiltration of M2 macrophages and delayed wound healing suggesting a dichotomous role for these bioactive molecules (170). PPAR-γ has been shown to facilitate the M1-M2 transition (150). PPAR-γ has also been shown to play a role in MRSA clearance in chronic wounds by forming a glucose-depleted, hypoxic, fibrotic abscess that hinders bacterial growth (150). Finally, Guo et al. have demonstrated that AGEs contribute to excessive macrophage autophagy, which polarizes macrophages towards an M1 phenotype and supports sustained inflammatory processes (171). Moving forward it will be critical to understand how bacterial bioburden and composition contributes to the activation/inactivation of these pathways and to better understand the downstream consequences of dysregulated M1-M2 phenotypic switching in humans.

### Other Modifiers of Host Antibacterial Immune Responses in the Wound Microenvironment

Aging is among one of the most significant predisposing factors to delayed healing and chronic wound formation. Older individuals are more commonly affected by vascular disease, venous insufficiency, unrelieved pressure, and post-surgery wound complications (172). Further, various studies have shown that aging affects all stages of the healing process including delayed re-epithelialization, angiogenesis, and collagen deposition (173–175). Changes skin strength may also result in a more pronounced breakdown of skin epithelial barriers, which may increase the bacterial bioburden in the wound microenvironment (175, 176). At the level of the immune system, advanced age is associated with a hyperinflammatory state (177). Innate cells have delayed infiltration, reduced phagocytic capacity, decreased reactive oxygen and nitrogen species production and impaired intracellular killing (178, 179). Interestingly, a recent study observed no age-related changes in TLR2 and FcγRIII expression, phagocytosis, and bactericidal activity in aged mice with cutaneous *S. aureus* infection (157). However, they did find that neutrophils had diminished sensitivity to chemokines (e.g., KC, MIP-2, and MCP-1), which reduced their chemotaxis into the wound bed and delayed healing (157). Additional studies are required to evaluate if similar dysfunction is observed in humans.

Diabetes is also associated with delayed healing and chronic wound formation (**Table 4**). Among the most common manifestations are non-healing foot ulcers (180). In these individuals wound healing is influenced by a predisposition to vascular disease and neuropathy, hypoxia, and hyperglycemia (181). Of particular importance, impaired vascular flow creates a prolonged hypoxic wound microenvironment, which along with hyperglycemia, contributes to oxidative stress (181). Hyperglycemia is also associated with AGE formation, which further delays healing (182). Chronic low-grade inflammatory also defines diabetes pathology and dysregulated healing response in these individuals (183). Emerging evidence suggests altered inflammatory responses to bacteria in the wound microenvironment may contribute to the development of these chronic wounds. In diabetic mice with cutaneous *S. aureus* infection, there is excessive macrophage and neutrophil infiltration into the wound but poor localization to abscess (148). These mice also have altered inflammatory cytokine and chemokine profiles during early and late stages of infection. Despite forming large abscesses, their structure had diffused immune cell organization and higher bacterial burdens (148). In mice infected with *P. aeruginosa*, diabetes was associated with prolonged M1 activation, which impaired healing processes by diminishing re-epithelialization, granulation tissue formation and angiogenesis (158). Alternatively, Nguyen et al. found diabetic mice inoculated with *S. aureus* biofilms had reduced TLR2 and TLR4 mRNA expression and high levels of inflammatory cytokines (IL-1β and TNF-α) (159). They also showed diabetic mice experience poor neutrophil penetration into regions with bacterial aggregates and downregulation of myeloperoxidase activity, a marker of neutrophil oxidative burst (159).

**Table 4:** Summary of other physiological factors that modify the inflammatory responses to bacteria in wound models.

Physiological State	Wound Model	Bacterial Species	Host Response	Effects on Wound Healing	Reference
Ageing	Mouse with full-thickness excisional wound	*S. aureus* Newman	No age-dependent changes in TLR2 expression, FcγRIII expression, phagocytosis, and bactericidal activity in macrophages and neutrophils ↓Neutrophil sensitivity to chemokines KC, MIP-2, and MCP-1 ↓Neutrophil chemotaxis and infiltration	↑Bacterial colonization, ↓Wound closure	(157)
Diabetes	Mouse full-thickness wound	*P. aeruginosa* ATCC27853	Prolonged M1 activation (TNF-α, IL-1β, IL-6) M2 activation (IL-10, arginase-1, or ym1)	↓Re-epithelialization ↓Granulation tissue formation ↓Angiogenesis ↓Wound closure	(158)
	Mouse full-thickness wound	*S. aureus* UAMS-1 biofilm	↓TLR2, ↓TLR4 mRNA expression ↓TNF-α, ↓IL-1β mRNA expression ↓Neutrophil infiltration in regions containing bacterial aggregates ↓MPO activity	↓Wound closure ↑Bacterial burden	(159)
Chronic venous leg ulcer (CVLU) or diabetic foot ulcer (DFU)	Wound exudate from patients with a CVLU or DFU	CVLU: *Pseudomonas, Staphylococcus, Corynebacterium* spp. DFU: *Corynebacterium, Staphylococcus* spp.	↑Bioburden (≥ 10^7^ CFU/ml), CVLU: ↑Angiogenin, ↑ICAM-1, ↑IL-1β, ↑IL-4, ↑IL-6, ↑TNF-α, ↑TNFr2, ↑VEGF, ↑antioxidant capacity DFU: ↓IFN-γ, ↓IL-2, ↓IL-4, ↓IL-5, ↓IL-12p40, ↓IL-12p70, ↓IL-13, ↓TGF-β1 CVLU vs DFU, CVLU: ↑IFN-γ, ↑IL-1β, ↑IL-2, ↑IL-4, ↑IL-13, ↑TNF-α, ↑VEGF, ↑collagenase activity DFU: ↑carbonyl, ↑malondialdehyde, ↑antioxidant capacity	NA	(160)
	CVLU biopsy	*P. aeruginosa* (N=5) and *S. aureus* (N=5) aggregates	↑neutrophil infiltration in *P. aeruginosa* infected wounds compared to *S. aureus* infected wounds	NA	(161)
Recurrent subcutaneous SSSI	Mouse subcutaneous wound	MRSA USA300 LAC	Innate immune memory provides protection against recurrent SSSI: ↑M1 macrophages, ↑LDCs, ↑NK cells, ↑Th17 cells, ↑neutrophil influx to abscess ↑total macrophage population in inguinal lymph nodes. ↑IL-22, ↑IFN-γ, ↑IL-17A, ↑IL-6 ↑MIG, ↑RANTES in the skin and ↑IP-10 in blood AMPs ↑CRAMP, ↑mβD-3	↓Abscesses ↓Bacterial burden	(162, 163)

NA, not applicable; KC, keratinocyte-derived chemokine; Ym1, Chitinase-like 3 protein; MPO, myeloperoxidase; SSSI, skin and soft tissue infection; LDC, Langerhans^+^ dendritic cell; NK, natural killer; MIG, monokine inducible by IFN-γ; RANTES, regulated upon activation, normal T cell expressed and secreted; IP-10, interferon gamma-induced protein 10; AMP, antimicrobial peptide.

The type of chronic wound and the diversity/number of bacteria found in the wound may also play an important role in determining the magnitude of the inflammatory responses (**Table 4**). For example, chronic venous leg ulcer (CVLU) exudate commonly contains *Pseudomonas, Staphylococcus, Corynebacterium* spp (160). With increasing levels of bacteria (≥10^7^ CFU/ml), angiogenin, ICAM-1, IL-1β, IL-4, IL-6, TNF-α, TNFr2, VEGF, and antioxidant capacity are shown to be elevated (McInnes et al., 2014). Alternatively, diabetic foot ulcers, which lacked *P. aeruginosa*, have diminished IFN-γ, IL-2, IL-4, IL-5, IL-12p40, IL-12p70, IL-13, and TGF-β1 production with increasing bacterial bioburden (160). In comparison of the inflammatory response between CVLUs and DFUs, McInnes et al. determined that CVLUs have higher levels of IFN-γ, IL-1β, IL-2, IL-4, IL-13, TNF-α, VEGF, and increased collagenase activity compared to DFUs. On the other hand, DFUs showed higher levels of carbonyl, malondialdehyde and antioxidant capacity compared to CVLUs (160). Moreover, Fazli et al. found in chronic venous leg ulcer biopsy samples, *P. aeruginosa* aggregates displayed amplified neutrophil infiltration compared to *S. aureus* aggregates in wounds, suggesting that these differences may be due to the intrinsic properties of *P. aeruginosa* to mount a higher inflammatory response (161). Additional studies are required to further explore these associations and differences.

Past exposures to bacterial infections may also alter local immune responses to wound infections (**Table 4**). Upon first exposure to a pathogen, innate immune cells can adapt, such that upon re-exposure, they mount a heightened pathogen-specific inflammatory response to boost host defense and provide long-term protection (184). This phenomenon is known as innate immune memory (184). Emerging evidence suggests innate immune memory may provide protection against recurrent staphylococcal skin infection. In mice primed by prior S. aureus infection, lesion severity was reduced by increased M1 macrophage, Langerhans+ DCs (LDC), NK cells, Th17 cells, and neutrophil influx to the abscess (162, 163). Interestingly, cytokines IL-6, IL-17, IL-22, chemokines MIG and RANTES, and antimicrobial peptides CRAMP and mβD-3 contributed to the development of innate immune memory in these mice (162, 163). In the context of chronic wounds, innate immune cells are chronically exposed to bacterial biofilms rather than a first exposure followed by recurrent exposure paradigm. Whether persistent activation of innate immune cells constrains development of this protective innate immune memory or not remains to be elucidated.

## Targeting Host-Pathogen Interactions to Restore Healing Processes

Managing and treating chronic wounds can be very challenging. It requires a comprehensive wound assessment and the establishment and implementation of a plan of care. These individualized plans aim to optimize the local wound environment and drive healing using four basic strategies: wound cleansing, debridement, moisture control, and bacterial balance (35). Among these, controlling bacterial bioburden is essential for wound healing and can be done by 1) reducing the levels of bacteria found in the wound and/or by 2) optimizing host immune responses to the infection. For the purposes of this review, we will briefly discuss how standard treatments reduce bacterial bioburden and/or restore immune function. Then, we will discuss emerging therapeutics designed to target interactions between bacteria/biofilms and the host immune response to restore healing processes.

### Debridement and Negative Pressure Wound Therapy (NPWT)

Both debridement and NPWT have been shown to affect bacterial bioburden and/or inflammation in the wound bed. Many types of debridement technologies exist including biological (maggot/larval therapy), mechanical, hydrosurgical, chemical, autolytic, enzymatic, surgical, and conservative sharp debridement (35). The purpose of these procedures is to remove necrotic or infected tissue to facilitate healing. In addition to removing infected tissue, debridement has been shown to remove and disrupt mature biofilms. Wolcott et al. found serial debridement to continually remove mature biofilms can be used to increase the efficacy of topical antimicrobials on newly forming/immature biofilms, which are more susceptible to treatment (185). NPWT, also known as vacuum assisted closure (VAC) therapy, improves healing by removing excess exudate, maintaining moisture balance, and increasing blood flow into the wound. It has also been shown to control infection and modulate immune responses. In animal models, NPWT has been shown to have anti-biofilm effects (186–188). It has also been shown to modulate growth factor, cytokine expression, and matrix metalloproteinases to support healing (189, 190). This includes decreasing IL-6, iNOS, TNF-α, IL-1β, MMP-1, and MMP-9 and upregulating VEGF, TGF-β1 and TIMP-1 in patients with diabetic foot ulcers (191, 192).

### Antiseptics

Irrigation solutions such as sterile normal saline or sterile water are the simplest wound cleansing methods. Antiseptic agents, such as octenidine dihydrochloride (OCT), polyhexamethylene biguanide (PHMB), povidone-iodine, and super oxidized hypochlorous acid (HOCl) and sodium hypochlorite (NaOCl) are widely used in topical wound therapy in solution form or as functionalized dressings due to their high microbicidal and anti-biofilm properties (193–197). OCT and PHMB have surfactant properties to help break apart biofilms and PHMB is particularly useful due to its low toxicity (198). Silver- and copper- impregnated dressing are also widely used in chronic wound management (199, 200). However, a recent scoping review by Rodriguez-Arguello et al. established mixed results in terms of antimicrobial activity and clinical effectiveness of silver agents (201). Moreover, a recent systematic review that evaluated the efficacy of commercially available topical agents containing silver, iodine, PHMB, or hypochlorous acid concluded that a lack of *in vivo* evidence makes it difficult to make recommendations for biofilm-infected wounds (202). Little research is available on the effects of these agents on modulation of the immune/host response. In human ex vivo full-thickness skin injury, OCT has been shown to dampen pro-inflammatory and anti-inflammatory cytokines IL-8, IL-33, and IL-10, but not growth factors VEGF and TGF-β1 (203). In an ex vivo porcine skin model, povidone-iodine, silver lactate, and OCT showed antiprotease activity that was dependent on their wound penetration ability (204). These anti-inflammatory properties need to be further investigated *in vivo*.

### Antibiotics

Antibiotics are often also used in the management of chronic wound infections. However, the type of antibiotic prescribed, and the administration route depend on the clinician evaluation and must take into consideration the microbial bioburden, patient clinical condition (e.g., allergies, immunocompetence, comorbidities, and pregnancy), the severity of the infection, and drug toxicity and dosage (35, 205). For instance, contaminated and colonized wounds do not require the use of antibiotics to improve wound outcomes. Alternatively, local infection often involves the use of topical antimicrobials including antibiotics compared to systemic infections that use systemic antibiotics (35, 206).

Topical antibiotics provide a high drug concentration at the infection site and possess low toxicity since the body systemically absorbs a low amount of drug. Moreover, they are easy to apply, and their use can avoid the use of systemic antibiotics. However, topical antibiotics cannot be prescribed to treat deep tissue infections, can affect healing, can cause hypersensitivity, and can select for resistant microorganisms (207, 208). They are often formulated as ointments, gels, creams, and powders, and only a few are available for use (e.g., bacitracin, fusidic acid, gentamicin, mafenide acetate, metronidazole, mupirocin, neomycin, nitrofurazone, polymyxin B, retapamulin, silver sulfadiazine, sulfacetamide Na^+^). The antibiotic used also depends on the type of wound. Bacitracin, neomycin sulfate, and polymyxin B are frequently used in combination to treat minor skin injuries. Silver sulfadiazine cream is commonly used as a topical antibiotic to treat DFUs and pressure ulcers (209, 210). Gentamicin and sulfacetamide are used to treat secondary infections, colistin (polymyxin E) is used for MDR gram-negative infections and metronidazole is commonly used to treat infections caused by anaerobic microbes and to reduce the odor of wounds (207).

Systemic antibiotics are used in patients with more severe infections. However, in these cases, the resistance profile of the pathogen is closely related to the success of the treatment (206). For instance, vancomycin is the first-line treatment to fight MRSA infections, followed by second-line agents, including linezolid, daptomycin, and quinupristin-dalfopristin. Other examples of systemic antibiotics used to treat chronic wounds include macrolides (e.g., erythromycin, azithromycin, and dirithromycin), β-lactams (e.g., cephalosporin, amoxicillin), penicillinase-resistant penicillins (e.g., cloxacillin, oxacillin), trimethoprim-sulfamethoxazole, fluoroquinolone, tigecycline, and clindamycin (35, 206, 211). The type of antibiotic prescribed is also dependent on the type, location and severity of the wound. For mild and moderate DFUs, narrow-spectrum antibiotics are recommended, especially those active against Gram-positive cocci (211). Alternatively, severe DFUs should be initially treated with broad-spectrum antibiotics, such as carbapenem β-lactams or the combination of β-lactam antibiotics and β-lactamase inhibitors (e.g., piperacillin/tazobactam, ampicillin/sulbactam, ticarcillin/clavulanic acid and amoxicillin/clavulanic acid) (210). The use of intravenous antibiotics is recommended to treat pressure ulcers when there is sign of osteomyelitis (209). In these cases, antibiotics that penetrate the bone are required, such as β-lactams (e.g., penicillin and cephalosporin), fluoroquinolones aminoglycosides, and glycopeptides (e.g., vancomycin), linezolid, and rifampin (212).

Several challenges, such as the formation of multi-species biofilms, are implicated in antibiotic treatment success. Wounds are often infected with polymicrobial biofilms formed by several species of resistant bacteria. These biofilms are commonly resistant to topical and systemic antibiotics, which reduces the effectiveness of the antimicrobial treatment. For instance, Shettigar et al. found the authors showed that 60% of the DFU samples investigated were infected with polymicrobial biofilms, in which the isolated *E. faecalis* showed higher resistance to antibiotics than non-biofilm grown cells (213). Furthermore, bacteria within biofilms produce several protective components. Among them, the EPS matrix is an important factor that impairs the penetration of antibiotics into the wound bed. For instance, *P. aeruginosa* EPS contains extracellular DNA and alginate lyase that impairs the diffusion of aminoglycosides (214, 215). Another problem associated with the low permeability of antimicrobial agents through the biofilm structure is the induction of resistance due to the low concentration of antibiotics when they reach the bacterial cells (56).

### New Approaches to Treating Chronic Wounds

**Figure 3:**
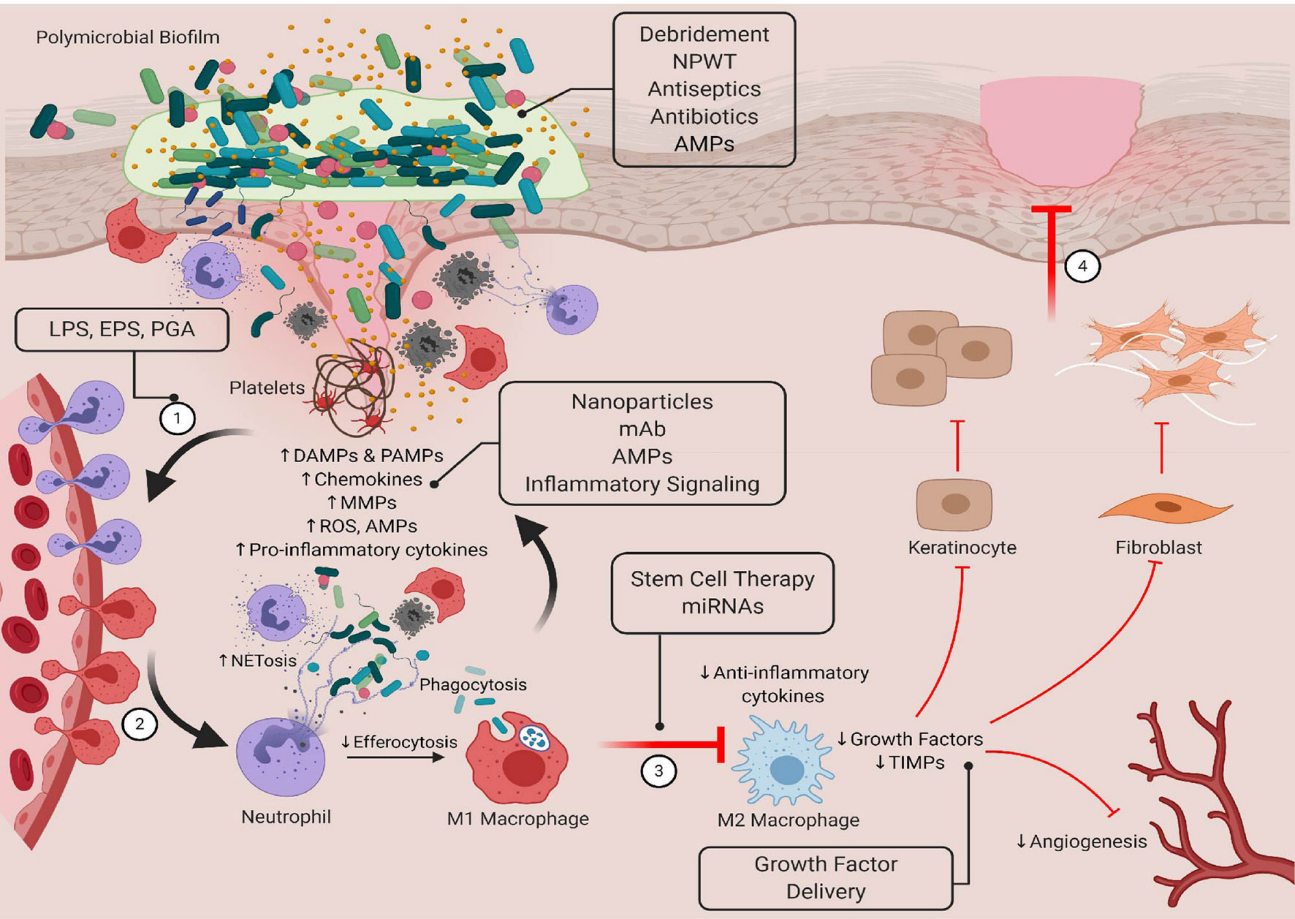
Targeting bacteria-innate immune interactions to restore healing in chronic wounds. Standard therapies such as debridement, NPWT, antiseptics, and antibiotics have been shown to reduce bacterial bioburden in the wound bed, but they do not always restore healing processes. New therapeutics that have both antimicrobial and immunomodulatory properties may be able to overcome the limitations of more traditional treatments. Here, we show novel therapeutics that target these interactions that can be used in early and late stages of healing to restore tissue homeostasis. LPS, lipopolysaccharide; EPS, extracellular polymeric substance; PGA, peptidoglycan; AMP, antimicrobial peptide; mAb, monoclonal antibody; miRNA, microRNA. Created with BioRender.com.

A variety of new treatments are under development to improve healing and restore tissue homeostasis. Among the most promising are candidates that target or work in conjunction with the innate immune system to improve antibacterial immune responses and/or regulate inflammatory responses. Some of the most promising are highlighted in **Figure 3**. These pathways and molecules represent viable targets because they can be used to modulate both early and late healing processes. There is also a reduced risk of developing drug resistance. Here, we will discuss the potential use of antimicrobial peptides in targeting bacteria levels/biofilm formation and in modulating immune function. We will also describe other strategies under development that seek to develop smarter and controlled innate immune responses by priming the antibacterial immune responses, restoring inflammatory balance, and selectively inducing an M1-M2 transition.

### Antimicrobial Peptides

Antimicrobial peptides (AMPs) show tremendous potential for the treatment of severe and chronic infections. These peptides have a broad-spectrum antibacterial activity that usually involves attacking multiple hydrophobic and/or polyanionic targets (216). They have been shown to induce pore formation, disrupt cellular and organelle membrane integrity, inhibit protein and nucleic acid synthesis, block enzymatic activity, inhibit cell wall synthesis, and induce apoptosis through the generation of ROS (217, 218). In addition to their antimicrobial effects, AMPs also modulate immune response to improve bacterial killing by increasing numbers of antigen presenting cells, facilitating the release of NETs, enhancing of phagocytosis, modulating dendritic cell differentiation and T cell activation, suppressing inflammatory signaling and anti-inflammatory cytokines (218). Further, many AMPs have been shown to promote wound healing by modulating of keratinocyte cytokine production and migration, re‐epithelialization and angiogenesis (219). In addition, several natural and synthetic AMPs exhibit strong antibiofilm activities, for example by disrupting bacterial communication networks (quorum sensing), inhibiting bacterial cell adhesion or by stimulating biofilm dispersal (220). Here, we discuss just a few examples of these bioactive molecules.

In humans, AMPs are produced by a variety of cell types including skin epithelia cells. In conditions of health, RNase 5, RNase 7, psoriasin and calprotectin are produced and have antimicrobial activity against both Gram-negative and Gram-positive bacteria (221–223). Alternatively, under conditions of inflammation or infection, β-defensins (h-BD) and LL-37 are selectively induced to mount a wide spectrum of antimicrobial activities including antibiofilm activities (219, 224). In addition to their microbicidal activity, hBD has been shown to regulate inflammatory processes by inhibiting TLR signaling pathways and transcriptionally repressing of pro-inflammatory genes expression (225, 226). Further, hBD-3 has also been shown to act as a ligand for the macrophage receptor CCR2, attracting macrophages to sites of epithelial injury (227). LL37 has also been shown to neutralize the activation of macrophages *via* LPS and induce proliferation and migration of endothelial cells (228). It also contributes to multiple phases wound repair including the stimulation endothelia cells and fibroblasts (228, 229) stimulation of keratinocytes (230), neovascularization (228) and angiogenesis (231). A number of approaches are currently under development for administering hBD and LL-37 including poly(vinyl alcohol)/cellulose acetate (PVA/CA) films (232), nanoparticles (233, 234), and nanostructured lipid carriers (235). Further, the efficacy of LL-37 cream in treating DFU is currently in clinical trials (https://clinicaltrials.gov).

Interestingly, wound healing is a relatively conserved evolutionarily process and several species including insects such as *Drosophila*, *Caenorhabditis elegans* and amphibians have been shown produce their own AMPs. For example, Pseudin-2 isolated from the frog *Pseudis paradoxa* has been shown to have a broad-spectrum antimicrobial potency and skin biocompatibility against multidrug-resistant (MDR) *Pseudomonas aeruginosa* (236). It has also been shown to facilitate infected-wound closure by reducing inflammation through suppression of interleukin-1β (IL-1β), IL-6, and tumor necrosis factor alpha (TNF-α) (236). Alternatively, the synthetic peptide A3-APO, derived from natural insect products, has shown promise in *in vivo* models. It was shown to efficiently ameliorate resistant aerobic and anaerobic intradermal infections, in part by increased recruitment of epithelial macrophages and their immunomodulatory/anti-inflammatory effects (237). Epinecidin-1 (Epi-1), an AMP derived from grouper *Epinephelus coioides* is also of potential interest. This molecule has been shown to have antibacterial, antifungal, and antiviral activity *in vitro* and *in vivo* (238). In mice with MRSA, Epi-1 has been shown to decrease levels of TNF-a, IL-6, and MCP-1, while also regulating monocyte recruitment during wound healing (239). It also enhances wound closure and angiogenesis (239). These molecules and many others are in early stages of development but represent promising antimicrobial and immunomodulatory therapeutics.

### Jump-Starting Innate Immune Responses Using Bacteria/Bacterial Components

Topical application of PAMPs isolated from bacteria has been used to stimulate wound healing by initiating inflammatory processes in early stages of healing. In mice, Kostarnoy et al. have shown that application of lipopolysaccharide (LPS), a component of the outer membrane of Gram-negative bacteria and a potent endotoxin, improves healing by accelerating the resolution of inflammation by increasing macrophage infiltration, the expression of proinflammatory cytokines (IL-6, IL-1β, and leukemia inhibitory factor (LIF)), CC-chemokines (CCL2, CCL7, CCL3 and CCL5), growth factors (VEGF, TGF-1β, and FGF-2) in the wound microenvironment and by increasing collagen synthesis in the wound microenvironment (240). Similarly, the exopolysaccharide or extracellular polymeric substance EPS-S3 derived from the marine bacterium *Pantoea* sp. YU16-S3 has been shown to be a potential biomolecule to promote skin tissue regeneration (241). *In vitro*, EPS-S3 has been shown to increase dermal fibroblasts and keratinocytes, and macrophage activation (241). In vivo, EPS-S3 increases expression of growth factors and adhesion molecules HB-EGF, FGF, E-cadherin suggesting this exopolysaccharide may modulates wound healing through the Wnt/β-catenin pathway (241). Interestingly, other studies have shown that rats subcutaneously implanted with PVA sponges inoculated with non-viable S. aureus or its peptidoglycan have improved healing responses (242, 243). These responses are associated with increased macrophage, neutrophil and fibroblast infiltration, collagen production, and angiogenesis, which contribute to the formation of reparative tissue.

### Improving Antimicrobial Responses

Another area of interest is to develop therapeutics that enhance antimicrobial immune responses. Yu et al. have developed iron oxide nanoparticles (IONPs) that are taken up by macrophages to enhance bactericidal activity against intracellular S. aureus. They do this by increasing the expression of pro-inflammatory M1 markers iNOS, IL-1β, and TNF-α and amplifying ROS production (244). They also found these bactericidal effects could be enhanced by coupling IONPs with vitamin C in a Fenton reaction to augment the formation of ROS in the form of hydroxyl radicals (244). Alternatively, Okumura et al. shown that the pharmacological agent AKB-4924 can promote antibacterial immune responses by stabilizing HIF-1α (245). Intracellular HIF-1α accumulation heightens pro-inflammatory responses by increasing expression of LL-37 and IL-8 in human monocytes and enhancing bactericidal activity *in vitro* and *in vivo* (245).

### Targeting Inflammatory Balance

As described above, persistent or sustained inflammation is a central drive of dysregulated healing and chronic ulcer formation. Therapeutics that neutralize pro-inflammatory biomolecules such as cytokines, chemokines and bioactive lipids are under investigation to limit tissue damage and restore timely healing processes. Among these, Song et al. have examined the effect of anti-TNF- α monoclonal antibody (MAb) in a primate model of S. aureus-associated skin (246). Systemic administration of anti-TNF-α MAb reduced abscess severity through suppression of circulating proinflammatory IL-8 and IL-12, benefiting host responses to bacterial challenge (246). Alternatively, Brandt et al. have shown that therapeutics that target the LTB4/BLT1 signaling axis can reduce abscess severity and inflammation by limiting neutrophil recruitment, improving chemotaxis, and restoring chemokines and cytokine levels (148). More recently, Vågesjö et al. have developed CXCL12-delivering Lactobacilli to topically administer chemokines with increased bioavailability for wound healing processes (247). In mice models, sustained topical application of CXCL12 increased proliferation of TGF-β expressing macrophages (247) and accelerated wound healing in healthy mice, mice with hyperglycemia, and peripheral ischemia, and in an *in vitro* human skin disk model (247).

Growth factors also play a critical role in modulating inflammation and inducing cell proliferation, angiogenesis, and granulation tissue formation during wound healing. Diminished levels of growth factors, such as VEGF and FGF-2 have been associated with chronic pressure ulcers (248). To restore this imbalance novel therapeutics have been focused on delivering growth factors to the wound bed to promote healing. In a study on diabetic foot ulcer patients receiving intralesional epidermal growth factor (EGF) therapy, García-Ojalvo et al. demonstrated a reduction in systemic proinflammatory biomolecules C-reactive protein (CRP), IL-6, soluble FAS (sFAS), and CCL3, as well as oxidative capacity and nitrosilative (nitrite/nitrate) stress biomarkers (249). Further, intralesional EGF therapy was shown to increase soluble RAGE (sRAGE), which may have protective effects in diabetic patients (249). Another approach to increase growth factors is to provide bone-marrow mesenchymal stem cells (BM-MSCs). Bai et al. developed an injectable hydrogel made from crosslinking N-chitosan and adipic acid dihydrazide with hyaluronic acid-aldehyde to deliver BM-MSCs into the wound bed (250). *In vitro* and *in vivo* investigations in diabetic wound healing showed that the hydrogel was able to stimulate BM-MSC-derived secretion of TGF-β1, VEGF, and FGF-2 as well as inhibiting chronic inflammation through M2 macrophage polarization (250). It also induced granulation tissue formation, collagen deposition, tissue vascularization, and improved wound closure (250). The LeucoPatch uses a similar approach to promote healing. This circular patch is comprised of fibrin, white cells and platelets derived from the patient’s own blood, which concentrates cells and growth factors (e.g., PDGF, TGF-β, EGF and VEGF) to support healing. In an observed-masked randomized controlled trial, it was found to improve healing outcomes and shortened time to healing (251).

### Promoting Macrophage Polarization Towards an M2 Phenotype

Given that M1/M2 macrophages play a key role in regulating wound healing, reprogramming macrophage polarization towards an M2 tissue repair phenotype represents an attractive target in later stages of the healing process. Stem cell therapy has emerged as a promising treatment for modulating these processes. In a mouse diabetic wound model, treatment with hyaluronic acid spongy hydrogels impregnated with neurogenically conditioned human adipose stem cells (hASCs) increased neoepidermial thickness and accelerated wound closure (252). Moreover, addition of hASCs in comparison to hydrogel treatment alone increased the M2/M1 macrophage ratio, suggesting that hASCs can promote the transition to the repair phase of healing (252). A recent systematic review by Raghuram et al., identified adipose-derived stem cells, bone marrow-derived stem cells, bone marrow-derived mononuclear cells, epidermally derived mesenchymal stem cells, fibroblast stem cells, keratinocyte stem cells, placental mesenchymal stem cells, and umbilical cord mesenchymal stem cells being used *in vitro* and *in vivo* as potential treatments for chronic wounds, however, clinical effectiveness still requires investigation due to heterogeneity of wound etiology (253).

A multitude of micro-RNAs (miRNAs) have been associated with each phase of wound healing, from pro-inflammatory cytokine signaling to proliferation and remodeling, and offer a potential therapeutic strategy for the treatment of chronic wounds (254). Saleh et al. developed adhesive hydrogels containing miR‐223 5p mimic loaded hyaluronic acid-based nanoparticles (255). In vitro, miR-233 5p had the ability to polarize M1 macrophages towards an M2 phenotype, with increased expression of anti-inflammatory gene Arg-1, and suppression of proinflammatory cytokines TNF-α, IL-1β, and IL-6 (255). In vivo experiments on a mouse wound model, miR-233 5p was able to promote tissue vascularization and accelerate wound healing (255).

## Discussion

Ageing and immobile individuals as well as those with co-morbid conditions such as diabetes are at high risk for developing non-healing or chronic wounds. These wounds reduce quality of life and increase pain levels, risk for infection and prolong hospital stays. Chronic wounds are also difficult to treat and represent a significant financial burden on all healthcare systems. Globally, as the size of these populations grow, there is an urgent need to understand the pathophysiology of delayed wound healing and to develop effective therapies that repair tissue damage. Critical for the development of these new therapeutics is a comprehensive understanding of the cellular and molecular mechanisms underpinning bacteria-innate immune interactions.

There is still a lot to learn about the microbiological and immunological processes underlying bacteria-innate immune interactions in chronic wounds and their relative contributions to delayed healing. From the microbiological perspective, the use of molecular methods, such as RNA sequencing, has allowed for the identification of a larger diversity bacterial species in the wound. These methodologies have also been used to elucidate microbial activities, behaviors, strategies, and processes during infections (12). However, these approaches are associated with a substantial demand for financial, time and bioinformatic support and cannot be readily transferred into the clinic. Further, they cannot distinguish between living, dead or dormant bacteria and might overlook minority species (52). Moving forward, it will also be critical develop more standardized sampling and analysis to ensure reproducibility across studies (256–258). It is also important to note that, to date, most studies have been performed over an acute timeframe with the longest being over a 28-day period (75). Considering that chronic wounds can take up to 12 months to heal (259), or may not heal at all (55), there is little information about how bacterial populations change over longer time frames. We believe combining single cell analyses (transciptomics, flow, etc.), advanced microscopy and other techniques will provide critical insights into how biofilm structures as well individual cells contribute to chronic wound formation and chronicity across diverse microenvironments and patient populations. Further, we believe more long term longitudinal *in vivo* studies with larger samples sizes and standardized sample collection/analysis are urgently needed to fully understand the importance of microbial diversity, biofilms and the wound microbiome in chronic wounds infections and to elucidate the impact of aerobic, anaerobic, pathogenic and commensal bacteria in inflammation and wound healing across wound types.

To further our immunological understanding, we require clinically relevant model systems that mimic the complex, dynamic interplay between the wound microbiome, innate immune cells, and the various other factors that contribute to dysregulated healing in chronic wounds (260). To date, many studies have investigated interactions in the context of murine *S. aureus* abscess models. While *S. aureus* is a major causative agent of skin and soft-tissue infections, it is a specific type of skin infection. Other studies have evaluated how heat-killed bacteria and planktonic bacteria modulate immune responses but generally only evaluate short term and localized responses. Emerging research has started to evaluate the effects of single-species and polymicrobial biofilms on host immune responses *in vivo*. However, most of these studies have characterized differential responses to *S. aureus* and *P. aeruginosa* planktonic, single-species biofilm, and polymicrobial biofilms (69–71, 73, 74). Given that the wound microbiome is made of a wide diversity of bacterial species in polymicrobial biofilm communities, it is unclear how these findings can be translated into the clinical setting. We also think it is important to note, that much of what we know about the microbial diversity and immune responses in chronic wounds has been derived from models and clinical samples from patients with DFU. In this review, we found a few studies that evaluated interactions in other or non-DFU ulcers such as venous/arterial ulcers and pressure ulcers, but they were somewhat limited in scope (77, 160, 161). Future studies are required to evaluate how host immune responses are modulated by complex polymicrobial biofilms commonly found in wound beds and to better understand if these processes are affected by the wound type/tissue microenvironment.

## Author Contributions

All authors contributed to the article and approved the submitted version.

## Funding

The preparation of the publication was support by an International Research Seed Grant provided by Carleton University awarded to EC and JO. It was also supported by a Bruyere Research Institute Graduate Studentship Award awarded to ZV.

## Conflict of Interest

The authors declare that the research was conducted in the absence of any commercial or financial relationships that could be construed as a potential conflict of interest.
